# Supplementary figures and images for: Cellular response to small molecules that selectively stall protein synthesis by the ribosome
Source: PLoS Genet. 2019 Mar 15;15(3):e1008057. doi: 10.1371/journal.pgen.1008057 (PMC6436758; doi:10.1371/journal.pgen.1008057)

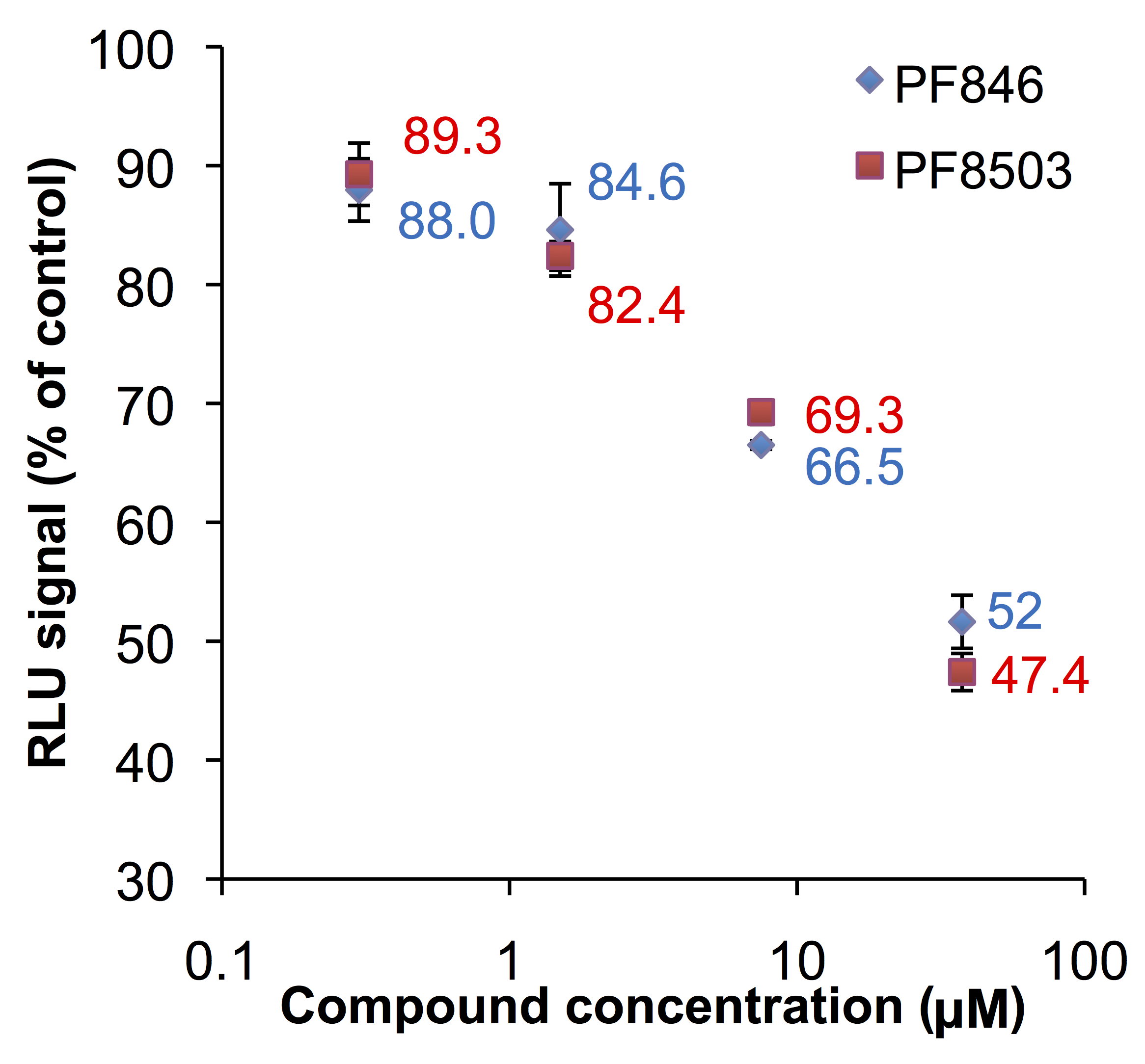

Supplement: S1 Fig — K562 cells were treated with various concentrations of PF846 or PF8503 in 96-well plates for 48 hr. Viability was measured as percentage of ATP signal in treated vs. not treated (control) wells. Shown are the are the mean and standard deviations of Relative Light Unit (RLU) measurements from 3 replicates each. (TIF) [file pgen.1008057.s001.tif]

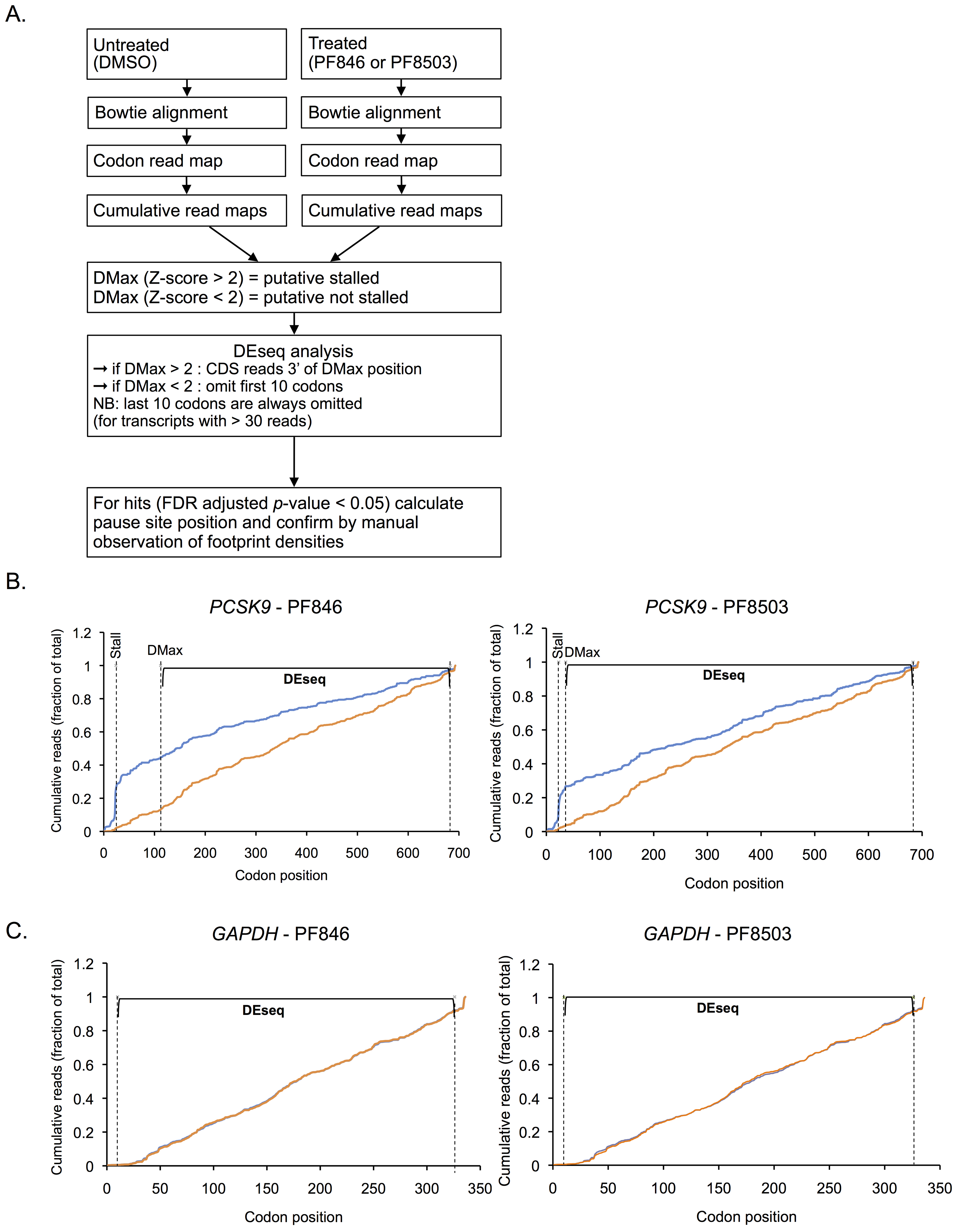

Supplement: S2 Fig — (A) Data processing flowchart, beginning with ribosome profiling libraries. (B) Examples of compound-induced stalling on PCSK9 mRNA, showing cumulative read maps, stall site positions, DMax positions, and regions used for differential expression (DEseq) analysis. (C) Example in which compound-induced stalling is not observed (GAPDH mRNA). Region used for differential expression analysis (DEseq) is marked. (TIF) [file pgen.1008057.s002.tif]

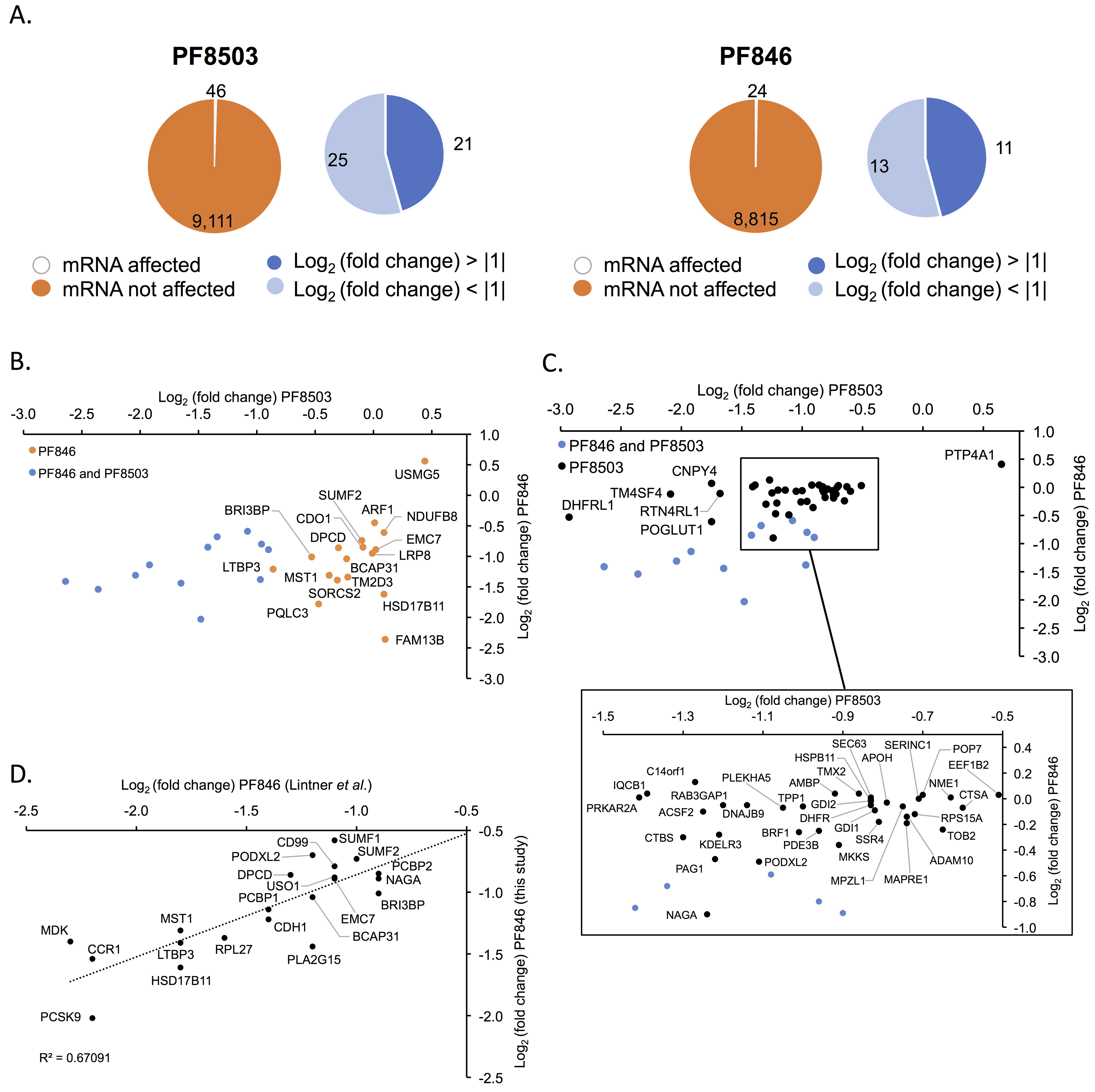

Supplement: S3 Fig — (A) Transcripts quantified from Huh-7 cells treated with 1.5 μM PF846 or 1.5 μM PF8503 for 1 hr before harvesting and ribosome protected RNA fragment library preparation. The log2(fold change) values correspond to the ratio of reads in compound-treated vs. control cells, summed 3’ of the DMax position, as described in the Materials and Methods and diagrammed in (S2 Fig). Number of mRNAs affected by PF846 or PF8503 (with adjusted p-value < 0.05, blue) among the total transcripts that could be analyzed (orange). Data are from three replicates, using the mean values of log2(fold change). (B-C) Values of log2(fold change) obtained from PF8503- or PF846-treated cells for shared PF8503 and PF846 targets (blue) and targets detected only in the PF846 treatment (orange) or only in the PF8503 treatment (black). Shared targets are also shown in Fig 1C. Data are from three replicates, with the mean values shown. (D) Comparison of the log2(fold change) values from PF846-treated cells obtained here with those from PF846-treated Huh-7 cells described previously [10]. (TIF) [file pgen.1008057.s003.tif]

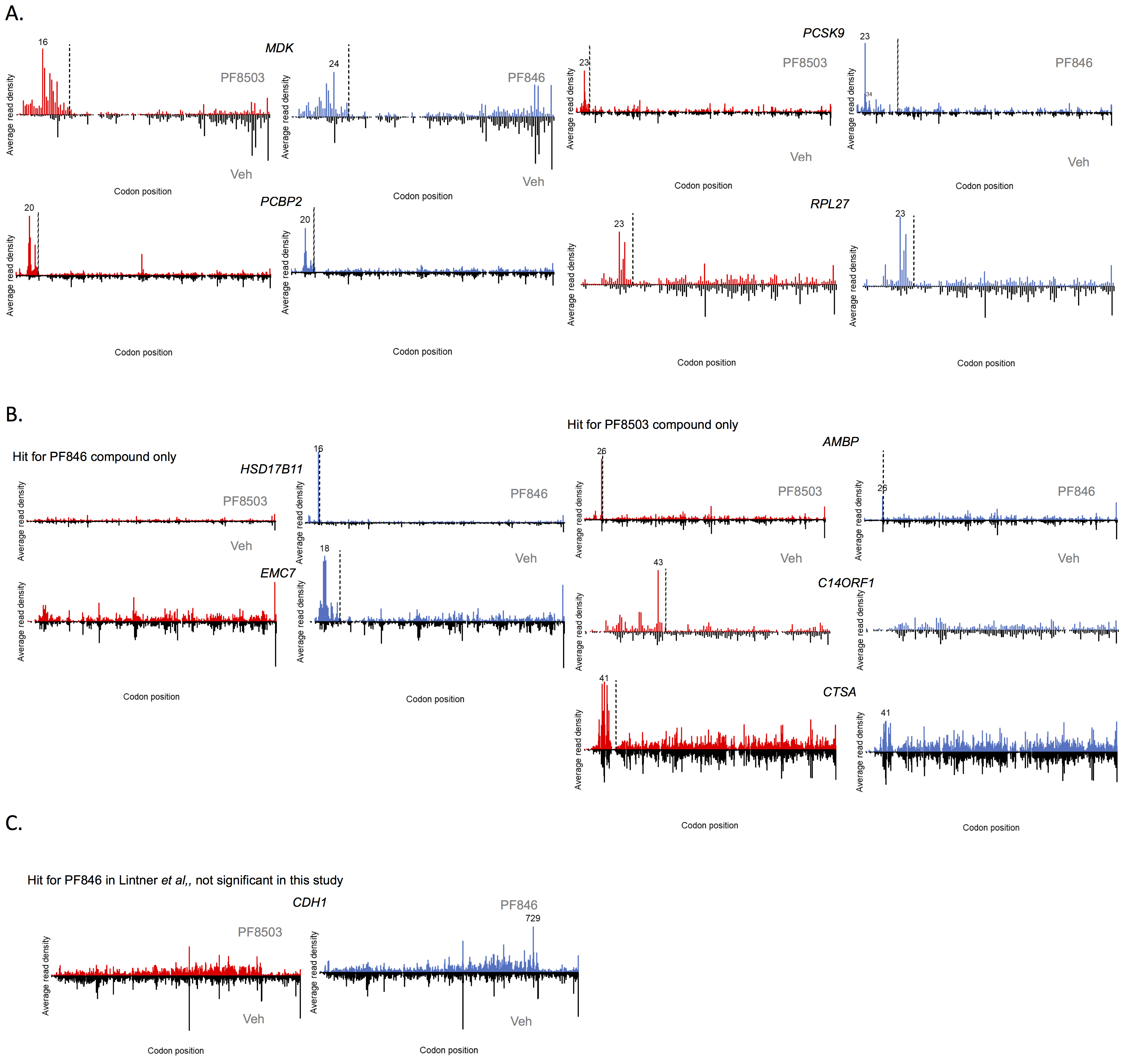

Supplement: S4 Fig — (A) Examples of transcripts impacted by both PF846 and PF8503. Average ribosome footprint densities along transcripts at codon resolution, from PF8503 (red), PF846 (blue) and Vehicle (DMSO, black) treated cells. The Vehicle profiles are shown as mirror images of the compound treatments. The main stall codon is marked for each condition and the dotted line represents the DMax position. (B) Example of transcripts impacted by only one compound. (C) CDH1 transcript showing a late stall only in the presence of PF846. Note, in the present experiments with PF846, CDH1 did not pass the DMax Z-score cutoff (S2 Table). In panels (A-C), the experiments were carried out in biological triplicate. (TIF) [file pgen.1008057.s004.tif]

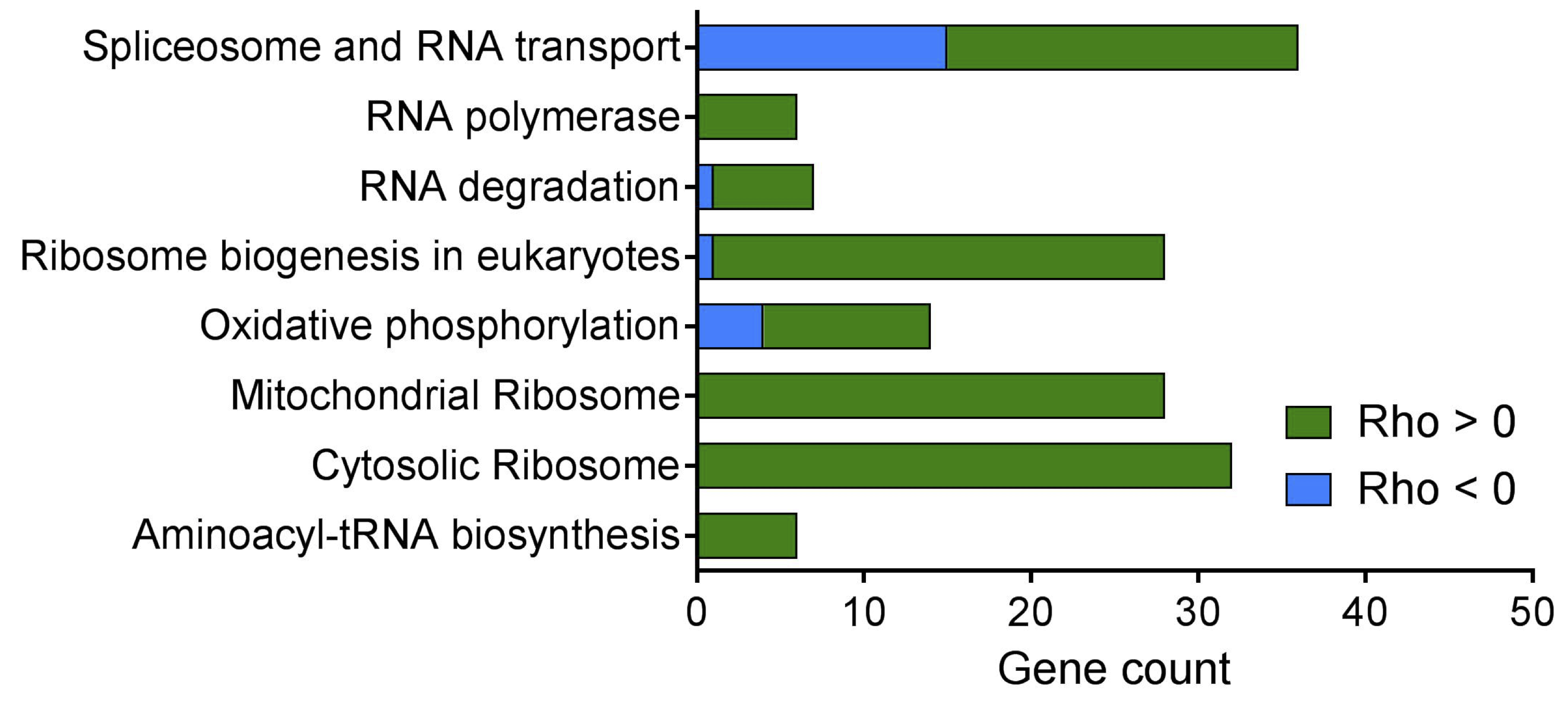

Supplement: S5 Fig — Pathways from STRING database analysis, with genes whose knockdown sensitizes (blue) or protects (green) cells from PF8503 toxicity. (TIF) [file pgen.1008057.s005.tif]

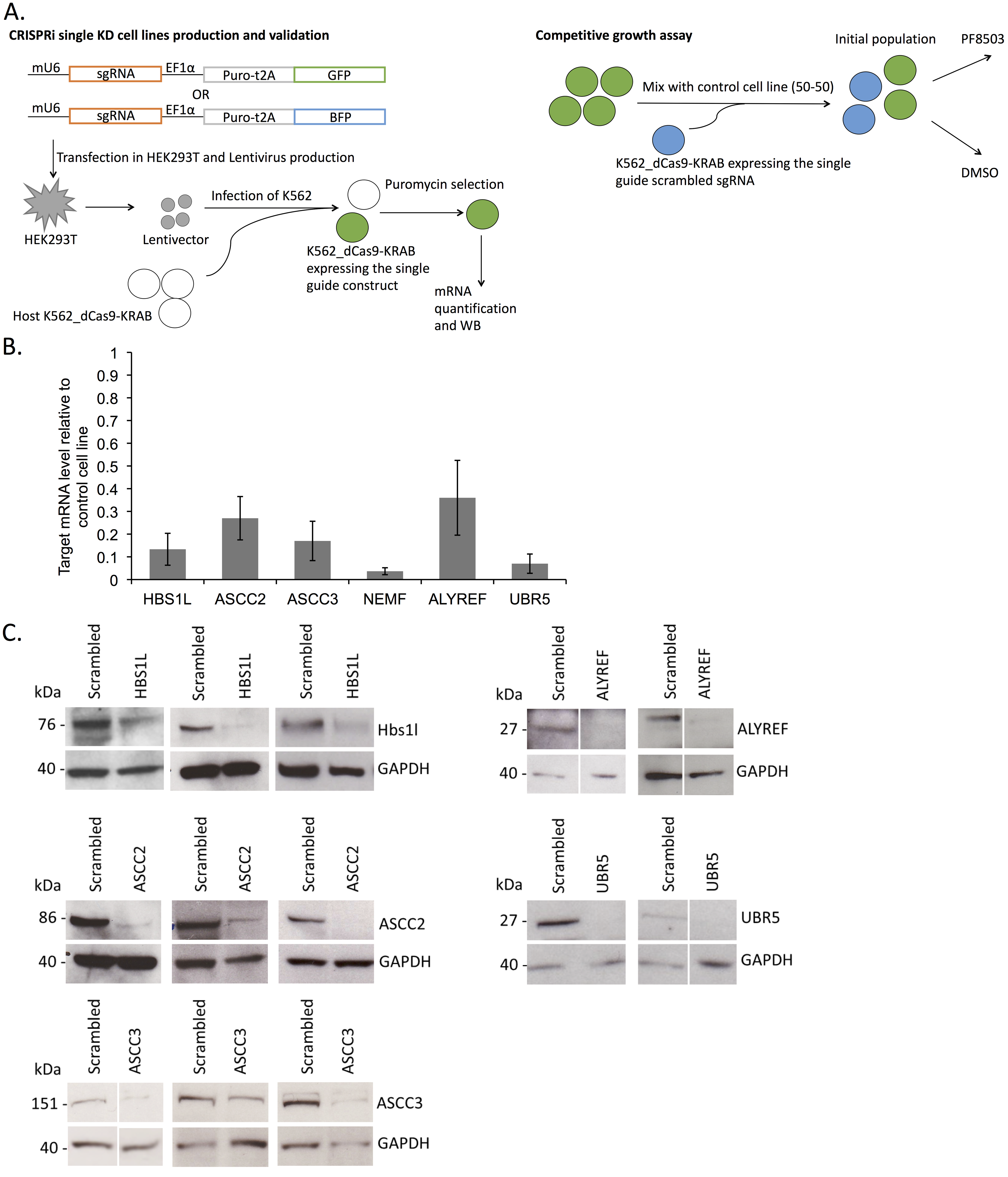

Supplement: S6 Fig — (A) Schematic of generation and validation of sgRNA-mediated knockdown in individual cell lines. Lentiviral vectors expressing puromycin resistance and BFP or GFP were used to ensure near-complete lentiviral infection. The resulting cell populations were used for RT-qPCR or Western blot analysis. (B) Levels of mRNAs for targeted genes, as determined by RT-qPCR. Measurements carried out in triplicate, with mean and standard deviation shown. (C) Western blots of proteins whose mRNA transcription was targeted by individual sgRNAs. Each Western blot is from cell lines used for triplicate experiments. (TIF) [file pgen.1008057.s006.tif]

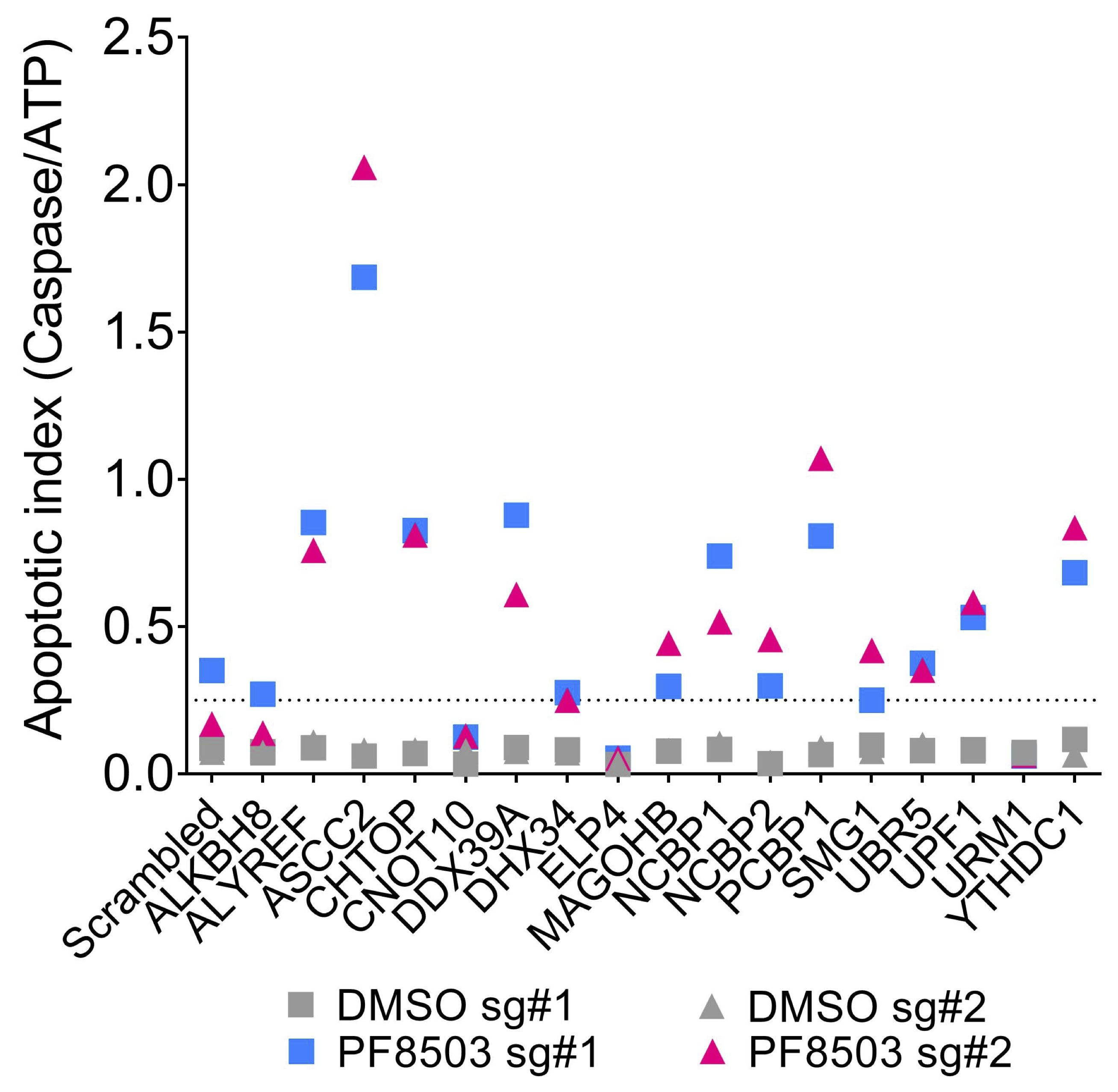

Supplement: S7 Fig — Survey of the apoptotic index (Caspase 3/7 levels divided by ATP levels) for cell lines expressing either of two different sgRNA targeting select proteins identified from the CRISPRi screen. Cells were incubated with 7.5 μM PF8503 for 6 days. (TIF) [file pgen.1008057.s007.tif]

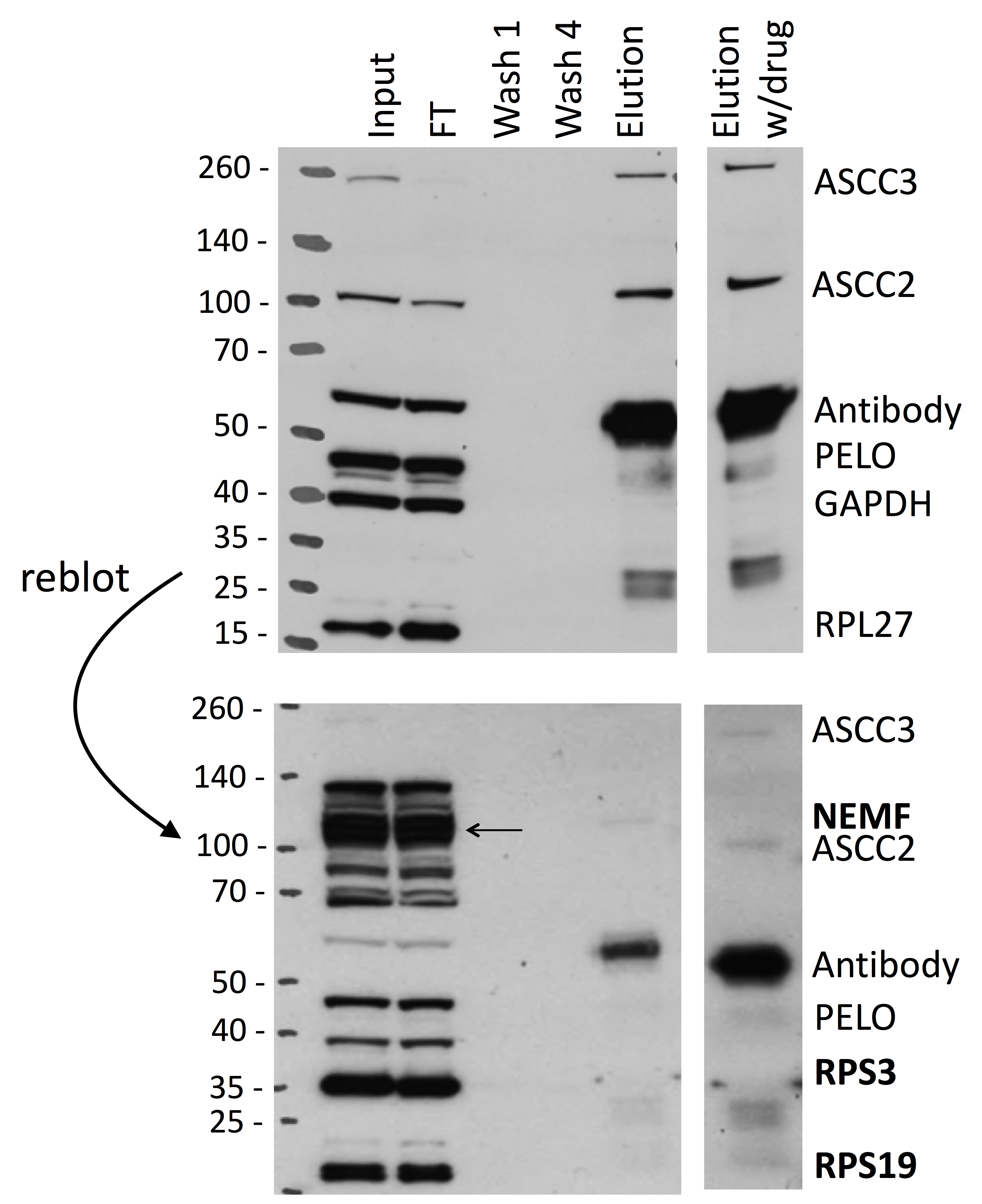

Supplement: S8 Fig — Full Western blot gels shown in Fig 3C. Top, blotted with antibodies against ASCC3, ASCC2, PELO, GAPDH, and RPL27. Bottom, membrane stripped and re-blotted for NEMF, RPS3, and RPS19 (bold). NEMF position is indicated by an arrow. (TIF) [file pgen.1008057.s008.tif]

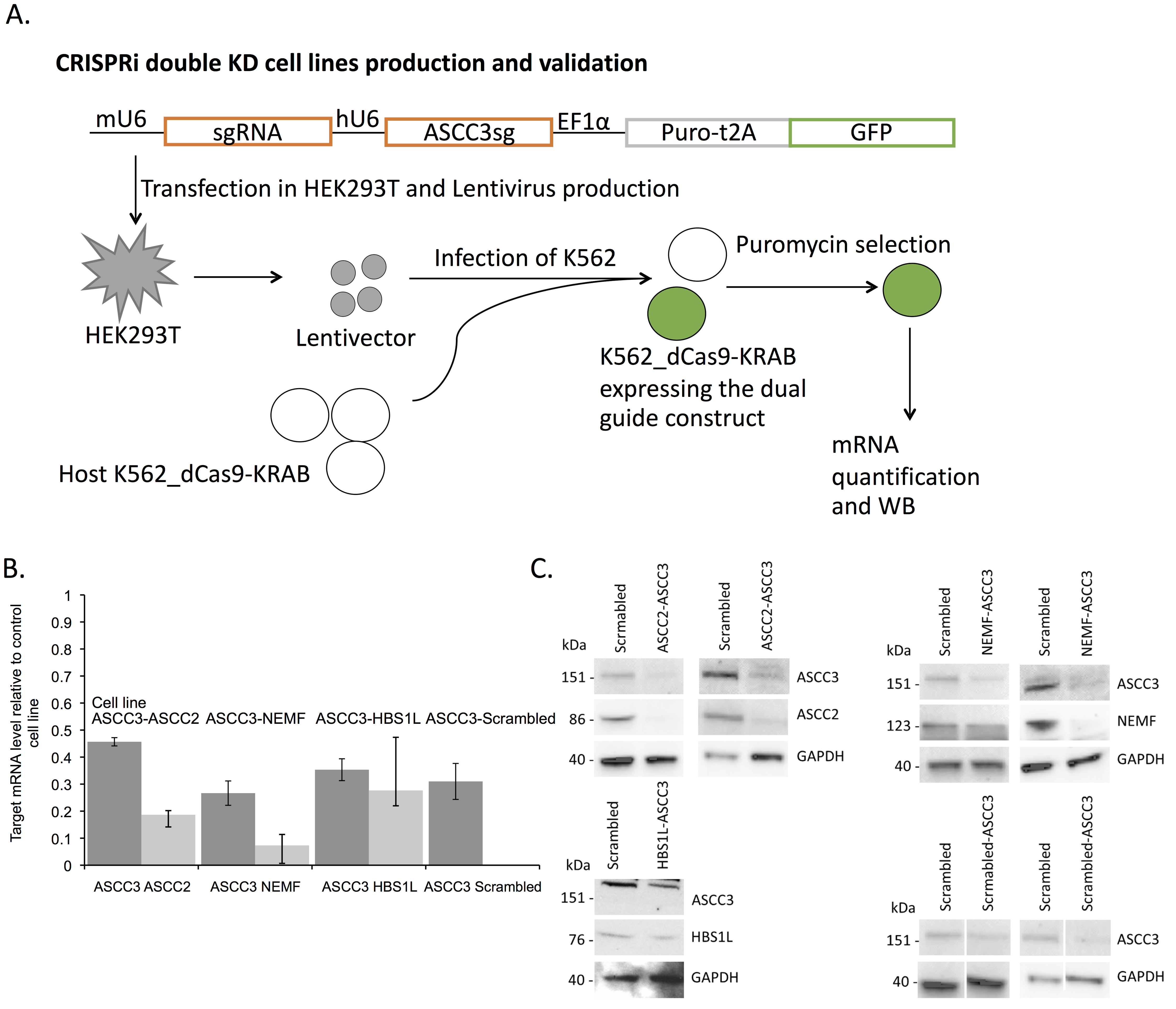

Supplement: S9 Fig — (A) Schematic of the construction of double knockdown cell lines. ASCC3 sgRNA expressed from the human U6 (hU6) promoter; second sgRNA expressed from the murine U6 (mU6) promoter. Puromycin resistance (Puro) and GFP expression were used to enrich lentivirally infected cells. The mRNA levels were determined using RT-qPCR, normalized to the housekeeping gene PPIA mRNA levels. (B) Target mRNA levels in double knockdown K562 cell lines expressing dCas9-KRAB and HBS1L, ASCC2, or NEMF sgRNAs along with ASCC3 sgRNA. Experiments carried out in triplicate. (C) Western blot analysis of corresponding protein levels in double knockdown cell lines, compared with cells expressing a scrambled guide RNA (NC, negative control). Blots were made using lysates from cells lines grown in triplicate. (TIF) [file pgen.1008057.s009.tif]

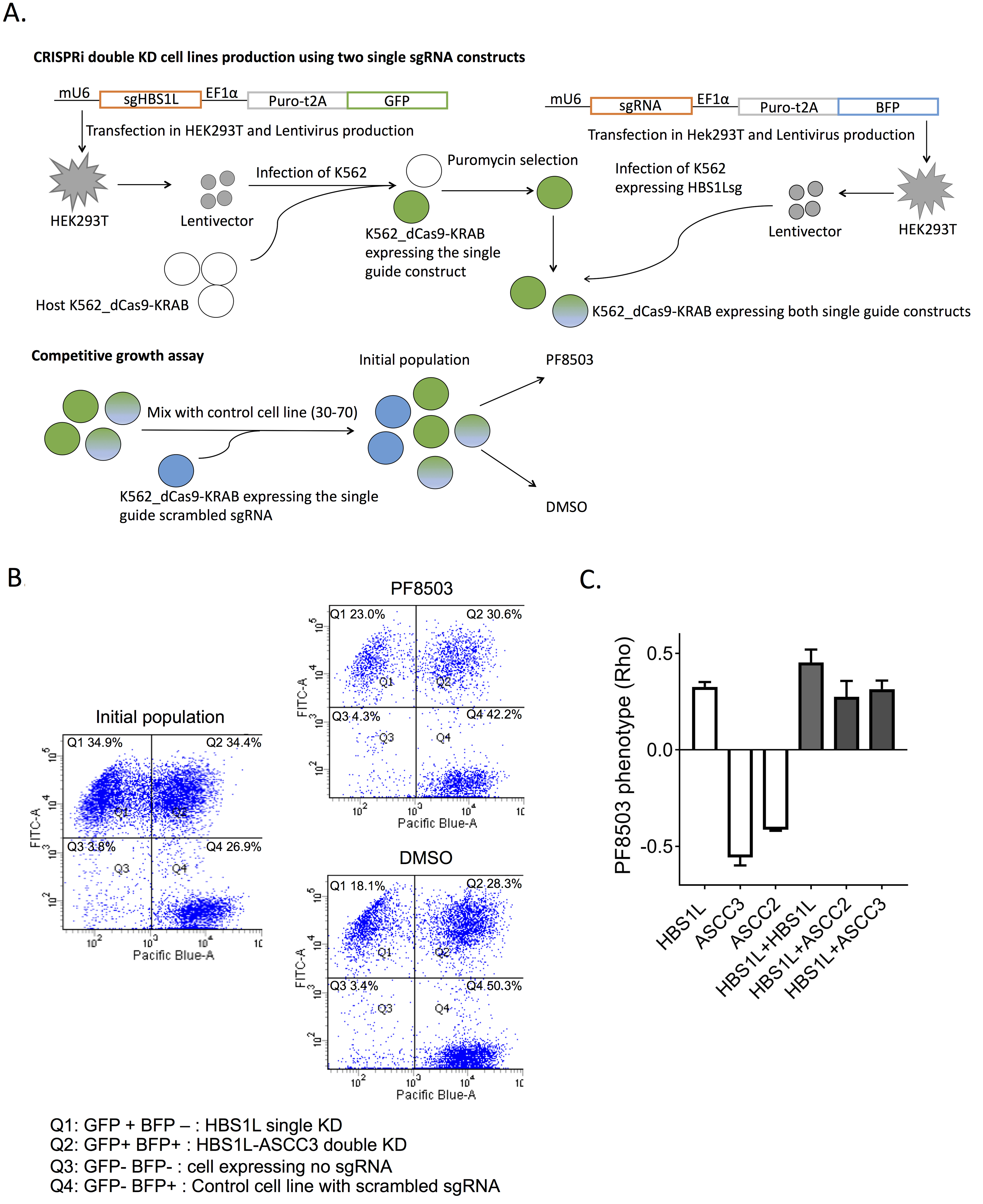

Supplement: S10 Fig — (A) Strategy used to generate double knockdown cell lines. Lentiviral vectors expressing single sgRNAs were used in serial infections to generate double-knockdown cells. Cells expressing sgRNA targeting HBS1L (HBS1L sg#2) with a GFP reporter were first validated for HBS1L mRNA knockdown and HBS1L protein knockdown (S6 Fig). These cells were then retransfected with a second lentivirus expressing an sgRNA targeting ASCC3, ASCC2, or HBS1L (HBS1L sg#1), with a BFP reporter. Populations of cells after Puromycin selection could then be scored for both GFP or BFP expression to indicate dual infection with the two lentiviruses. (B) Example FACS analysis of HBS1L-ASCC3 double-knockdown cells before and after selection in the absence or presence of 7.5 μM PF8503. (C) PF8503 toxicity phenotype (Rho) obtained from competitive growth assays in the presence of 7.5 μM PF8503 and scored using FACS analysis of GFP and BFP expressing cells as previously described [15,17]. Individual knockdown cell lines (open bars) and double knockdown cell lines (filled bars) are from experiments carried out in 2 replicates, from two independent transfections with mean and standard deviation shown. (TIF) [file pgen.1008057.s010.tif]

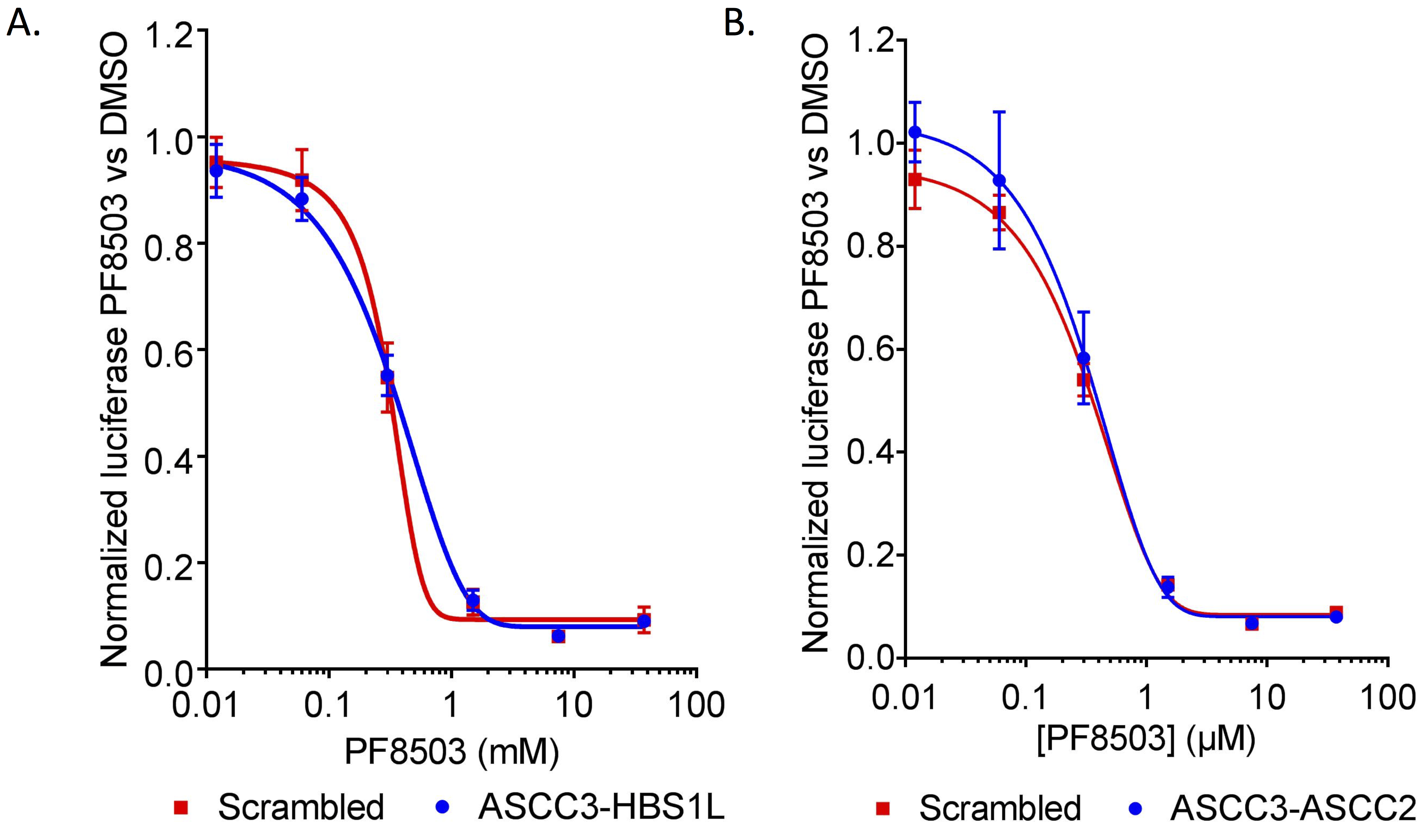

Supplement: S11 Fig — Effect of PF8503 dose on the relative signal of PCSK9(1–35)-Rluc and control Fluc mRNA reporters after 7–8 hr incubation in K562 double-knockdown cell lines. These cell lines were obtained by using single lentiviral constructs as shown in Fig 5A, with mU6-HBS1L-hU6-ASCC3 (A) or mU6ASCC2-hU6ASCC3 (B). Average of twelve (A) or six (B) experiments with standard deviation shown. (TIF) [file pgen.1008057.s011.tif]

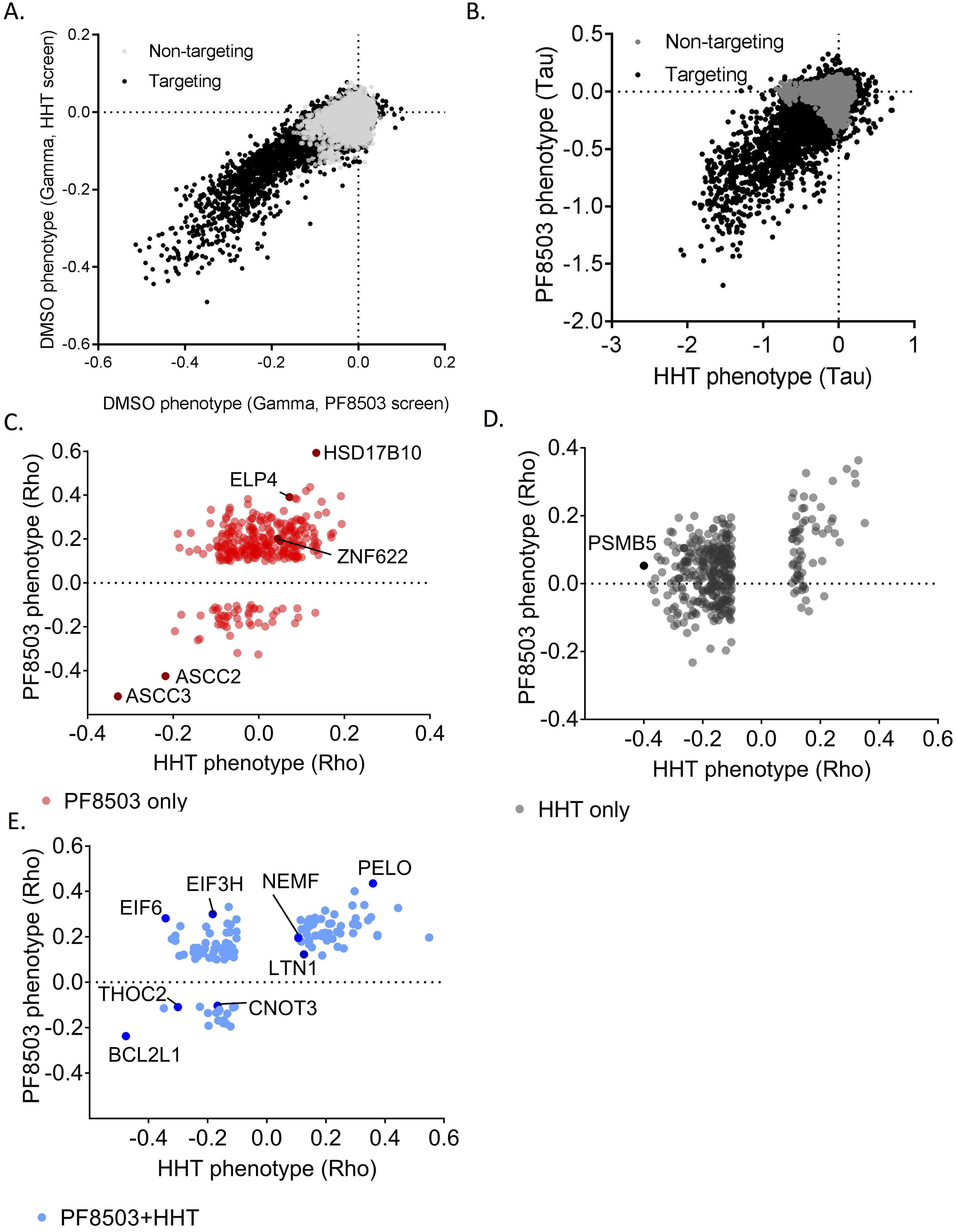

Supplement: S12 Fig — (A) Correlation between the untreated phenotypes (Gamma) obtained in the PF8503 and HHT screens, which were performed independently. (B) Correlation between the compound-treated phenotypes (Tau) obtained in the PF8503 and HHT screens. (C-E) Comparison of Rho phenotypes obtained in the HHT screen and in the PF8503 screen, for genes impacting cell fitness with PF8503 only (C), PF8503 and HHT (D), or HHT only (E). (TIF) [file pgen.1008057.s012.tif]
